# Current treatments for endometriosis in South Korea: an analysis of nationwide data from 2010 to 2019

**DOI:** 10.1038/s41598-023-36291-1

**Published:** 2023-06-13

**Authors:** Han Kyul Kim, Eun-San Kim, Kyoung Sun Park, Yoon Jae Lee, In-Hyuk Ha

**Affiliations:** 1 Jaseng Hospital of Korean Medicine, Gangnam-Daero, Gangnam-Gu, Seoul, Republic of Korea; 2 Jaseng Spine and Joint Research Institute, Jaseng Medical Foundation, Gangnam-Daero, Gangnam-Gu, Seoul, Republic of Korea

**Keywords:** Reproductive disorders, Health care

## Abstract

While a wide range of treatments, including medical therapies and surgery, are used to manage endometriosis, the characteristics and treatment status of patients who received these treatments have not been investigated in Korea. This study analyzed the Korean Health Insurance Review & Assessment Service—National Patient Sample (HIRA-NPS) data from 2010 to 2019 with 7530 patients diagnosed with endometriosis. Annual trends in the types of visit and surgery, medication prescriptions and associated costs were investigated. The analysis showed that surgery slightly decreased among the types of utilized healthcare services (2010: 16.3, 2019: 12.7), dienogest prescription rapidly increased due to national health insurance coverage from 2013 (2013: 12.1, 2019: 36.0), and the use of gonadotrophin-releasing hormone analogues decreased (2010: 33.6, 2019: 16.4). There was no significant change in total and outpatient costs per person over time. Regarding endometriosis treatment, conservative treatment mainly based on prescribed medications has been gradually replacing surgery. Particularly, the listing of dienogest for national health insurance coverage might have affected the trend. However, there were no significant changes in terms of total and medication costs per person.

## Introduction

Endometriosis is characterized by the presence of endometrium-like epithelium or stroma outside the endometrium and myometrium^1^. Symptoms of endometriosis include menstrual disorder, chronic pelvic pain, irregular uterine bleeding or infertility^2^. It is a benign sex hormone-dependent gynecological disease, usually associated with an inflammatory process^1,3^. The precise prevalence of endometriosis is unknown. A population-based study in the United States (U.S.) reported a decrease in endometriosis prevalence from 30.2 per 10,000 person-years in 2006 to 17.4 per 10,000 person-years in 2015, whereas its prevalence in Korean women increased from 21.2 per 10,000 persons in 2002 to 35.6 per 10,000 person-years in 2013^4,5^. Endometriosis may have an impact on the overall physical, mental, and social well-being of the patients; and despite extensive research, the etiology and pathogenesis of endometriosis remain unclear^6^. Guidelines by the Korean Society of Endometriosis, European Society of Human Reproduction and Embryology (ESHRE), Australia, Germany, and the Society of Obstetrics and Gynecology of Canada recommend the identification of endometriotic lesions with diagnostic laparoscopy, and where possible, confirmation with histology^1,7,8^.

Both medical and surgical therapies are used for endometriosis treatment^9^. For the management of endometriosis-associated pain, patients may be given non-steroidal anti-inflammatory drugs (NSAIDs) or other analgesics (either as monotherapy or in combination with other treatments)^10^. Additionally, female hormone therapy (contraceptives, progestogens, and gonadotrophin-releasing hormone [GnRH] agonists or antagonists) may be used^11^. Progestogens most commonly used for endometriosis treatment include medroxyprogesterone (MPA) and 19-nortestosterone derivatives (e.g., levonorgestrel, norethindrone acetate, and dienogest)^12^. Levonorgestrel-releasing intrauterine (LNG-IUS) system or levonorgestrel-releasing subdermal implant may also be considered as a treatment option^13^. Surgical intervention is a commonly used and clinically effective option for endometriosis management in patients who do not respond to or show intolerance to pharmacological treatment^14,15^. In a Cochrane meta-analysis of five randomized controlled trials that compared different laparoscopic surgical techniques with diagnostic laparoscopy only for endometriosis treatment, a significant improvement in pain outcomes was observed in the laparoscopic surgery groups^16^. Endometrial resection of endometriosis was also associated with improvement in pain^17^. For women who no longer wish to conceive, bilateral ovariectomy or hysterectomy is considered the most effective surgical intervention, and conservative procedures may be used if fertility preservation is desired^9,15^.

Endometriosis imposes a significant burden on health-related quality of life of women as well as on healthcare resources of national healthcare systems^18^. In a recent systematic literature review of studies published from 2000 to 2013, the estimated direct and indirect costs associated with endometriosis in the U.S. was $12,118 and $15,737 per patient per year, respectively^19^. A prospective, multi-center survey conducted in 10 European countries reported that the average annual total cost per woman with endometriosis in 2008, including costs of healthcare and productivity loss, amounted to nearly 10,000 euros^20^.

Investigating healthcare service utilization and costs for endometriosis treatment is expected to contribute to the establishment of clinical practice guidelines and policy making. In the U.S., comparisons of healthcare utilization and costs between women diagnosed with and without endometriosis and between patients who underwent endometriosis-related surgery and those without surgery revealed that that cost of surgery was the single largest contributor to the direct cost related to endometriosis treatment^21,22^. In Germany, changes from 2010 to 2019 were examined, and an increase in dienogest prescription was reported^23^. In Korea, details of healthcare service utilization and costs were not investigated in previous studies^8^. Therefore, this study analyzed the characteristics of patients diagnosed with endometriosis and their treatment status, including interventions and procedures, from 2010 to 2019 through the claims data of the Health Insurance Review & Assessment Service (HIRA) to provide reference data with useful information for the establishment of future healthcare policy on endometriosis treatment and for the selection of optimal treatment methods.

## Methods

### Data

This retrospective study used data of the Korean HIRA-National Patient Sample (HIRA-NPS) from 2010 to 2019. HIRA-NPS is cross-sectional database constructed by stratified sampling of all patients visiting medical institutions for healthcare services by gender and age group on a yearly basis. As of 2019, 2% of the total population in Korea has been sampled, and data from the previous year was re-sampled to match the 2% sampling rate. HIRA-NPS is a nationally representative health insurance database and passed the validity tests^24^. HIRA-NPS data include gender and age, diagnosis code, medical procedures and medications, treatment costs and demographic characteristics. This study was approved by the institutional review board (IRB) of Jaseng Hospital of Korean Medicine (JASENG 2022-10-012). The informed consent was waived by the IRB. This study was conducted according to the ethical principles stated in the Declaration of Helsinki.

### Population and characteristics

Endometriosis was defined based on the following diagnosis codes from the Korean Standard Classification of Diseases (KCD; eighth revision): N80.1, N80.2, N80.3, N80.4, N80.5, N80.6, N80.8, and N80.9 (N80.0, i.e., adenomyosis, was not considered). For each year, we extracted the data of all patients with primary diagnoses corresponding to these codes.

Patient ages were reported in 10-year increments. We considered a patient to have a comorbidity if they were diagnosed with it in at least once in the year for which their data were extracted. For comorbidities, infertility (N97), pelvic pain (R10), unspecified dysmenorrhea (N94.6), secondary dysmenorrhea (N94.5), uterine fibroids (D25) and Adenomyosis (N80.0) were included. We did not exclude patients based on their comorbidities because our aim was to investigate the current status of and trends in the treatments administered by clinicians for endometriosis.

### Measures

We analyzed all healthcare services (and associated costs) used by patients with endometriosis as their primary diagnosis. Data on outpatient visits, admission, surgery, and prescription were analyzed. Outpatient visits and hospital admissions are claimed differently, and the HIRA-NPS provides codes to differentiate between them. Surgery was defined as an operation for the genitourinary system among surgeries performed under the primary diagnosis of endometriosis. Laparoscopy surgery was also analyzed. All prescribed medications were investigated based on the Anatomical Therapeutic Chemical Classification System (ATC code). According to the guidelines, estrogen or progesterone, GnRH agonists, and NSAIDs, which are representative medications for endometriosis treatment, were investigated, and the most frequently used classes for each agent were reported. Codes used for definitions of procedures and medication were reported in Supplementary Table S1.

In medical costs analysis, the total costs spent yearly per person was extracted. Costs were categorized according to the healthcare services. The average cost of patients who used the service for each category was presented. Outpatient visits and admission costs included all expenses incurred during the visits to medical institutions due to the corresponding event. Surgery costs included all expenses incurred for the surgery. Medication costs were calculated according to the drug price.

### Analysis

The general patient characteristics are presented in terms of the number of patients (n) and percentage. All outcomes are presented according to each year of the study period and include the following: (1) prevalence of patients who used medical services (per 100 patients), (2) mean total number of visits or prescription days per 1 patient, and (3) mean total cost spent in 1 year per 1 patient. The prescription days and costs of medical services were calculated for patients who used those services; for instance, the total prescription days and costs of dienogest for 1 year were calculated for patients who were prescribed dienogest in that year.

Next, we examined whether the trend changed significantly over the years. We considered “the year” as a continuous variable and examined the linear changes accordingly. Outpatient visits and prescription days were considered count variables, whereas admission, surgery, and prescription rates were considered binary variables. Accordingly, these were analyzed using Poisson regression. For costs, we used a generalized linear model with log-link gamma distribution to address their skewedness. Findings from the crude analyses were considered the primary results of our study, because our research goal was descriptive^25^. We have also presented age-adjusted findings. Results of the Poisson regression analyses are presented as relative ratio (RR) per one year. For count variables, RR indicate changes in the probability of the event occurring for one more time or for one more day. For binary variables, RR indicate changes in the probability of the event occurring. Results of the gamma regression analysis are presented as the ratio change in the mean cost. P-value < 0.05 was considered significant. Subgroup analyses according to patient characteristics were conducted for two different age groups; ≥ 40 years of age and < 40 years of age.

## Results

### General characteristics of patients

From 2010 to 2019, there were 7530 patients with endometriosis in Korea. Table 1 outlined the general characteristics of patients by year. The number of endometriosis patients increased over the years (2010: 602, 2019: 1043). The age group with the highest proportion of endometriosis patients was 30–39 (2010: 37.4%, 2019: 37.8%). In terms of comorbidities, pelvic pain (24.3–31.6%) was the most frequently reported condition, followed by uterine fibroids (18.3–23.3%) and adenomyosis (10.4–15.4%) (Table 1).

**Table 1:** General characteristics of the patients.

Year (n)	2010	2011	2012	2013	2014	2015	2016	2017	2018	2019
	(602)	(639)	(651)	(660)	(711)	(721)	(797)	(809)	(897)	(1043)
Age group (%)										
0–19	13 (2.2)	10 (1.6)	8 (1.2)	13 (2.0)	8 (1.1)	12 (1.7)	11 (1.4)	9 (1.1)	9 (1.0)	15 (1.4)
20–29	162 (26.9)	177 (27.7)	165 (25.3)	149 (22.6)	191 (26.9)	178 (24.7)	206 (25.8)	220 (27.2)	237 (26.4)	264 (25.3)
30–39	225 (37.4)	245 (38.3)	276 (42.4)	279 (42.3)	285 (40.1)	274 (38.0)	290 (36.4)	340 (42.0)	326 (36.3)	394 (37.8)
40–49	168 (27.9)	172 (26.9)	168 (25.8)	186 (28.2)	208 (29.3)	222 (30.8)	248 (31.1)	204 (25.2)	273 (30.4)	305 (29.3)
50–59	27 (4.5)	28 (4.4)	29 (4.5)	29 (4.4)	15 (2.1)	32 (4.4)	35 (4.4)	34 (4.2)	44 (4.9)	54 (5.2)
≥ 60	7 (1.2)	7 (1.1)	5 (0.8)	4 (0.6)	4 (0.6)	3 (0.4)	7 (0.9)	2 (0.2)	8 (0.9)	10 (1.0)
Infertility	37 (6.1)	57 (8.9)	49 (7.5)	46 (7.0)	48 (6.8)	50 (6.9)	38 (4.8)	50 (6.2)	42 (4.7)	45 (4.3)
Pelvic pain	152 (25.2)	177 (27.7)	158 (24.3)	165 (25.0)	191 (26.9)	200 (27.7)	194 (24.3)	249 (30.8)	263 (29.3)	328 (31.4)
Unspecified dysmenorrhea	47 (7.8)	50 (7.8)	59 (9.1)	66 (10.0)	77 (10.8)	69 (9.6)	98 (12.3)	103 (12.7)	82 (9.1)	107 (10.3)
Secondary dysmenorrhea	19 (3.2)	15 (2.3)	20 (3.1)	19 (2.9)	14 (2.0)	12 (1.7)	29 (3.6)	25 (3.1)	28 (3.1)	24 (2.3)
Uterine fibroids	110 (18.3)	119 (18.6)	124 (19.0)	131 (19.8)	135 (19.0)	164 (22.7)	186 (23.3)	163 (20.1)	184 (20.5)	205 (19.7)
Adenomyosis	93 (15.4)	82 (12.8)	68 (10.4)	75 (11.4)	82 (11.5)	96 (13.3)	105 (13.2)	102 (12.6)	105 (11.7)	130 (12.5)

General characteristics of the patients were presented with numbers and percentages.

### Trend analysis

**Figure 1:**
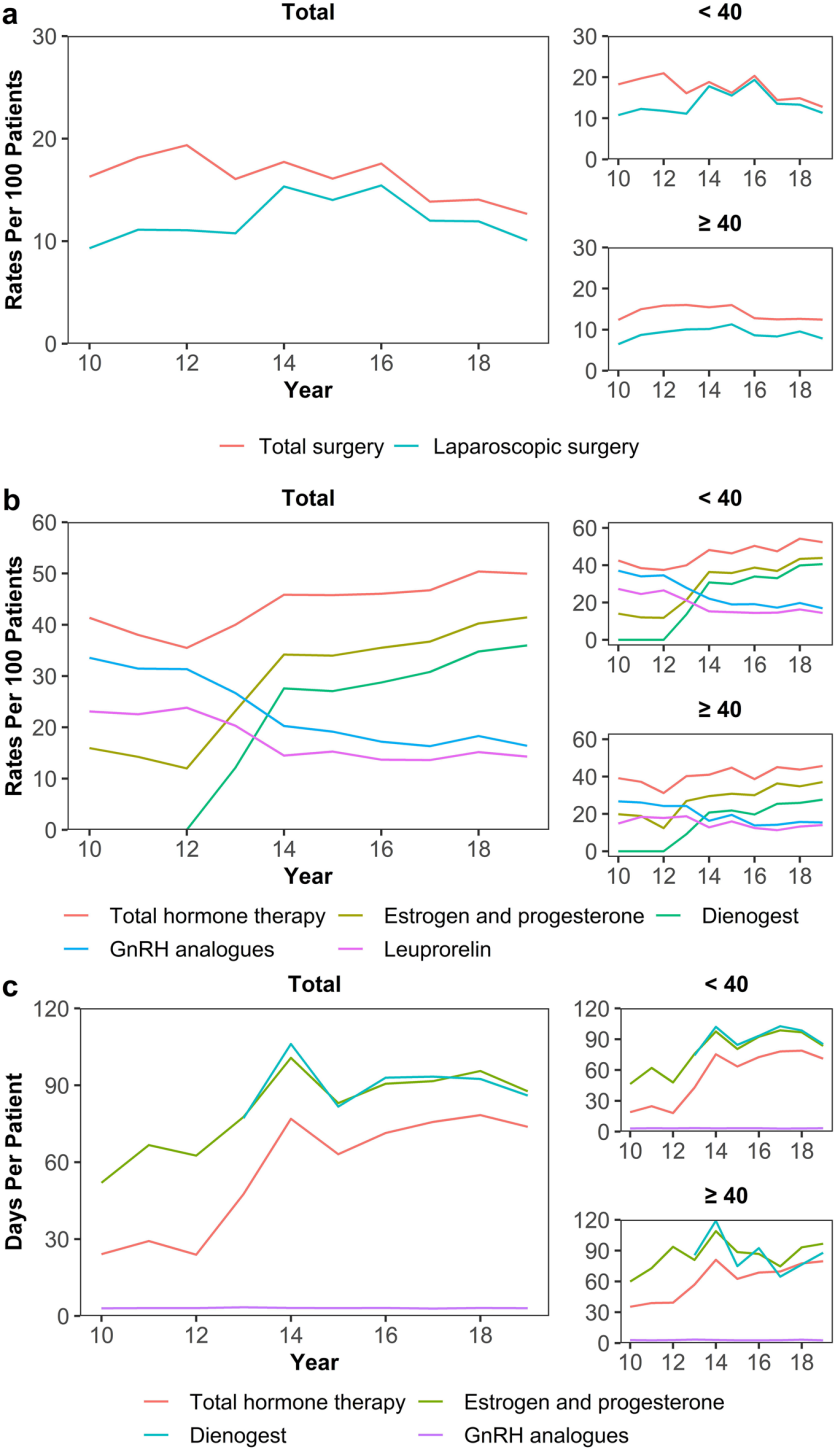
Trend analysis of surgery and prescription for endometriosis patients. (**a**) Surgery rates. (**b**) Prescription rates. (**c**) Prescription days. The values of total population and subgroup by ages (≥ 40 and < 40 years) were presented. Rates were expressed per 100 patients by year. Prescription days were provided with the mean (standard deviation) per one patient by year. Prescription days were calculated for patients who were prescribed corresponding medication. *GnRH* gonadotrophin-releasing hormone.

Treatments received by endometriosis patients and their changes over the years were presented in Table 2 and Fig. 1. The average frequency of outpatient visits was 2–3 times per year (2010: 2.39 ± 2.20, 2019: 2.82 ± 2.33), and the admission surgery rates showed a slight decrease (admission in 2010: 15.6, 2019: 12.7, RR 0.97 [95% CI 0.95 to 0.99]; surgery in 2010: 16.3, 2019: 12.7, RR 0.97 [95% CI 0.95 to 0.98]). In most cases, surgeries were performed with laparoscopy, and extirpation of benign adnexal tumor was the main type of surgery. The use of total hormone therapy increased (2010: 41.4, 2019: 50.0, RR 1.03 [95% CI 1.02 to 1.05]). Regarding the trend of medication prescriptions, the use of GnRH analogues (2010: 33.6, 2019: 16.4, RR 0.91 [95% CI 0.90 to 0.93]) and NSAIDs (2010: 26.6, 2019: 12.8, RR 0.89 [95% CI 0.87 to 0.91]) decreased. In contrast, the use of estrogen or progesterone sharply increased (2010: 15.9, 2019: 41.4, RR 1.13 [95% CI 1.11 to 1.14]), and particularly, the use of dienogest showed a rapid increase following the coverage of national health insurance from 2013 (2013: 12.1, 2019: 36.0, RR 1.27 [95% CI 1.25 to 1.30]). Since 2013, the prescription days of total hormone therapy had shown a sharp increase (2010: 24.1 ± 52.1, 2019: 73.8 ± 112.2, RR 1.11 [95% CI 1.11 to 1.11]). As a result of subgroup analysis by age, the decrease in surgery rate was only significant in those under the age of 40 years (β: − 0.64; RR:0.96 [95% CI 0.94 to 0.98]). While the use of total hormone therapy and dienogest increased in all age groups, the increase was greater in those under 40 years of age than their counterparts (total hormone therapy in patients < 40 years of age, RR 1.04 [95% CI 1.02 to 1.05]; total hormone therapy in patients ≥ 40 years of age, RR 1.03 [95% CI 1.00 to 1.05]) (Supplementary Tables S2, S3).

**Table 2:** The distribution of healthcare service utilization by year.

	2010	2011	2012	2013	2014	2015	2016	2017	2018	2019	RR (crude)	RR (adjusted)
Outpatient visits	2.39 (2.20)	2.48 (2.24)	2.75 (2.36)	3.08 (3.56)	2.97 (2.91)	2.89 (2.91)	2.95 (3.71)	2.86 (2.96)	2.97 (2.38)	2.82 (2.33)	1.01 (1.01 to 1.02)***	1.01 (1.01 to 1.02)***
Admission rate	15.6	17.7	18.0	14.1	15.9	14.7	17.1	13.1	13.6	12.7	0.97 (0.95 to 0.99)**	0.97 (0.95 to 0.99)**
Surgery rate	16.3	18.2	19.4	16.1	17.7	16.1	17.6	13.8	14.0	12.7	0.97 (0.95 to 0.98)***	0.97 (0.95 to 0.98)***
Laparoscopy	9.3	11.1	11.1	10.8	15.3	14.0	15.4	12.0	11.9	10.1	1.01 (0.99 to 1.03)	1.01 (0.99 to 1.03)
Surgery type												
Extirpation of benign adnexal tumor	9.3	8.9	10.0	10.0	11.4	9.6	11.4	9.4	9.4	8.4	0.99 (0.97 to 1.02)	0.99 (0.97 to 1.02)
Pelviscopic fulguration	1.2	1.4	0.8	1.5	2.0	1.8	1.5	1.2	1.1	0.5	0.96 (0.89 to 1.03)	0.96 (0.90 to 1.03)
Prescription rate												
Hormone therapy	41.4	38.0	35.5	40.0	45.9	45.8	46.0	46.7	50.4	50.0	1.03 (1.02 to 1.05)***	1.03 (1.02 to 1.05)***
Estrogen or progesterone	15.9	14.2	12.0	23.2	34.2	34.0	35.5	36.7	40.2	41.4	1.13 (1.11 to 1.14)***	1.13 (1.11 to 1.14)***
Dienogest	0.0	0.0	0.0	12.1	27.6	27.0	28.7	30.8	34.8	36.0	1.27 (1.25 to 1.30)***	1.28 (1.25 to 1.30)***
Tibolone	4.3	4.4	3.8	4.4	3.5	2.8	2.3	2.5	2.8	2.6	0.93 (0.89 to 0.97)***	0.93 (0.89 to 0.97)***
GnRH analogues	33.6	31.5	31.3	26.7	20.3	19.1	17.2	16.3	18.3	16.4	0.91 (0.90 to 0.93)***	0.91 (0.90 to 0.93)***
Leuprorelin	23.1	22.5	23.8	20.3	14.5	15.3	13.7	13.6	15.2	14.3	0.94 (0.92 to 0.95)***	0.94 (0.92 to 0.95)***
Goserelin	5.8	5.5	5.7	4.4	3.1	2.2	1.5	1.5	2.0	0.9	0.82 (0.78 to 0.86)***	0.82 (0.78 to 0.86)***
NSAIDs	26.6	28.5	26.9	17.3	13.4	10.7	12.5	11.1	10.7	12.8	0.89 (0.87 to 0.91)***	0.89 (0.87 to 0.91)***
Prescription days												
Hormone therapy	24.1 (52.1)	29.2 (59.2)	23.9 (50.6)	47.5 (84.4)	76.9 (111.4)	63.1 (97.4)	71.4 (117.2)	75.7 (113)	78.4 (119)	73.8 (112.2)	1.11 (1.11 to 1.11)***	1.11 (1.11 to 1.12)***
Estrogen or progesterone	52 (72.1)	66.6 (79.5)	62.5 (71.2)	77.7 (99.3)	100.7 (119.3)	83 (105.4)	90.6 (126.9)	91.6 (118.7)	95.6 (126.5)	87.6 (118.2)	1.03 (1.03 to 1.04)***	1.04 (1.03 to 1.04)***
Dienogest	–	–	–	77.1 (106.6)	106.1 (124.2)	81.7 (107.5)	93 (129.1)	93.4 (118.3)	92.5 (123.6)	86 (117.9)	0.99 (0.99 to 0.99)***	0.99 (0.99 to 0.99)***
Tibolone	53.3 (41.2)	88.9 (76.2)	87.1 (70)	116.1 (97.3)	85.5 (79.5)	98.3 (99.8)	92 (85.3)	102.3 (102.7)	108.4 (102.7)	100.5 (102)	1.04 (1.03 to 1.04)***	1.04 (1.04 to 1.04)***
GnRH analogues	3 (1.7)	3.1 (1.7)	3.1 (1.8)	3.4 (1.7)	3.1 (1.7)	3.1 (1.8)	3.1 (1.6)	2.9 (1.5)	3.1 (1.9)	3 (1.7)	1.00 (0.99 to 1.01)	1.00 (0.99 to 1.01)
Leuprorelin	3 (1.7)	2.9 (1.7)	3.1 (1.8)	3.4 (1.7)	3.1 (1.7)	3.1 (1.9)	3 (1.6)	2.8 (1.5)	3 (1.6)	3 (1.7)	1.00 (0.99 to 1.01)	1.00 (0.99 to 1.01)
Goserelin	2.4 (1.7)	3.3 (1.7)	2.7 (1.7)	3.2 (1.9)	3.2 (1.5)	2.8 (1.5)	3.4 (1.6)	3.4 (2)	2.9 (1.9)	3.4 (1.6)	1.02 (0.99 to 1.05)	1.02 (0.99 to 1.05)
NSAIDs	11.2 (17.6)	15 (30.9)	9.6 (10.6)	13.9 (38)	9.4 (11.8)	10.6 (15)	11.1 (18.9)	8.9 (12.3)	9.9 (13.3)	12.6 (23.4)	0.98 (0.98 to 0.99)***	0.98 (0.98 to 0.99)***

Outpatient visits and prescription days were provided with the mean (standard deviation) per one patient by year. Prevalence rates were provided with per 100 patients by the year. Prescription days were calculated for patients who were prescribed corresponding medication. Relative ratio (RR) was estimated with Poisson regression. We presented crude and age-adjusted RR per one year. RR of prescription days for dienogest was estimated with data after 2013. *P < 0.05; **P < 0.01; ***P < 0.001. GnRH, gonadotrophin-releasing hormone; NSAIDs, non-steroidal anti-inflammatory drugs. RR, relative ratio.

### Costs

There was no significant change in the total and outpatient costs per person over time. However, the costs of admission and surgery showed a gradual increase (admission in 2010: 1583.4 ± 486.2, 2019: 2482.1 ± 1032.4, cost ratio: 1.06 [95% CI 1.05 to 1.07]; surgery in 2010, 1506.8 ± 587.1; 2019, 2358.1 ± 1182.2, cost ratio: 1.06 [95% CI 1.05 to 1.07]). The cost of total hormone therapy showed a decrease over the years (total hormone therapy in 2010: 456.3 ± 351, 2019: 249.5 ± 181, cost ratio: 0.95 [95% CI 0.94 to 0.96]). The costs related to estrogen or progesterone increased, whereas those of GnRH analogues showed a sharp decrease (estrogen or progesterone in 2010: 21.8 ± 25.7, 2019: 183.4 ± 139.6, cost ratio: 1.15 [95% CI 1.13 to 1.17], GnRH analogues in 2010: 548.9 ± 312.2, 2019: 295.7 ± 174.4, cost ratio: 0.93 [95% CI 0.92 to 0.94]) (Table 3). In the subgroup analysis, the changes in total costs were non-significant for all subgroups. Other results were similar to the entire sample (Supplementary Tables S4, S5).

**Table 3:** The distribution of medical costs by year.

	2010	2011	2012	2013	2014	2015	2016	2017	2018	2019	Cost ratio (crude)	Cost ratio (adjusted)
Total cost	510.4 (801.1)	511.8 (757.2)	511.3 (793.8)	503.5 (863.1)	604.3 (959.3)	558.1 (910.1)	602.4 (983.9)	532.9 (924)	604.4 (1064.6)	529.9 (989.7)	1.01 (1.00 to 1.02)	1.01 (1.00 to 1.03)
Outpatient visits	270.4 (378.7)	260.5 (346.3)	212.1 (284.5)	221.6 (275)	250 (309.3)	232 (263)	231.9 (266.2)	230.4 (258.6)	263.3 (298.4)	221.3 (256)	0.99 (0.98 to 1.00)	0.99 (0.98 to 1.00)
Admission	1583.4 (486.2)	1478.7 (474.3)	1701.5 (458.4)	2031.1 (637.1)	2264.9 (463.4)	2268.8 (594.5)	2210.6 (754.8)	2341.1 (805.9)	2547 (1197.9)	2482.1 (1032.4)	1.06 (1.05 to 1.07)***	1.06 (1.05 to 1.07)***
Surgery	1506.8 (587.1)	1426.9 (552.1)	1554.8 (635.8)	1758.5 (826.6)	1999.8 (788.6)	2056.8 (849.3)	2089.6 (836.3)	2216.1 (942.8)	2439.9 (1170.5)	2358.1 (1182.2)	1.06 (1.05 to 1.07)***	1.06 (1.05 to 1.07)***
Hormone therapy	456.3 (351)	449.3 (342.4)	390.9 (276.1)	359 (263.9)	374.4 (312.3)	329.1 (230.2)	316.7 (237.2)	317.4 (222.4)	339.6 (228.4)	249.5 (181)	0.95 (0.94 to 0.96)***	0.95 (0.94 to 0.96)***
Estrogen or progesterone	21.8 (25.7)	30.9 (29)	23.3 (25.4)	144.5 (175.1)	281.8 (295.4)	253.8 (208.7)	253.9 (218.2)	268.3 (199.1)	278.1 (201)	183.4 (139.6)	1.15 (1.13 to 1.17)***	1.14 (1.12 to 1.16)***
Dienogest	–	–	–	253.3 (179.4)	343.7 (296.5)	311.8 (194.2)	308.5 (205.9)	314.4 (182.6)	317 (186.8)	207.9 (132.6)	0.95 (0.93 to 0.96)***	0.95 (0.93 to 0.96)***
Tibolone	22.3 (17.3)	32.0 (27.6)	25.5 (19.9)	31.6 (26.8)	22.4 (20.8)	25.4 (25.8)	23.1 (22.1)	24.0 (21.6)	23.7 (22.8)	18.6 (18.6)	0.97 (0.93 to 1.01)	0.96 (0.93 to 0.99)*
GnRH analogues	548.9 (312.2)	525.7 (308.6)	433.6 (253.7)	412.7 (238.9)	367 (227.7)	335.1 (203.8)	320.7 (193.9)	299.5 (197.9)	320.8 (201.9)	295.7 (174.4)	0.93 (0.92 to 0.94)***	0.93 (0.92 to 0.94)***
Leuprorelin	573 (309.4)	509.1 (304.5)	406 (220)	365.5 (181.5)	320.3 (191.1)	312.5 (182)	283.1 (150.3)	262.2 (137.3)	287.2 (145.2)	281.3 (156.8)	0.92 (0.91 to 0.93)***	0.92 (0.92 to 0.93)***

The costs were provided with the mean (standard deviation) per one patient by year. Costs were calculated for patients who used corresponding medical services. Cost ratio was estimated with generalized linear regression with log-link gamma distribution. We presented crude and age-adjusted cost ratio per one year. Cost ratio for dienogest change was estimated with data after 2013. *P < 0.05; **P < 0.01; ***P < 0.001. *GnRH* gonadotrophin-releasing hormone, *NSAIDs* non-steroidal anti-inflammatory drugs.

## Discussion

This study presented an analysis on the treatment status and costs for endometriosis using claims data of the national health insurance system for 10 years from 2010 to 2019. The comparisons of healthcare service utilization of endometriosis patients for the aforementioned period showed that the number of patients increased over the years, with the largest proportion of patients in the age group of 30–39 years. The surgery rate decreased, whereas the prescription of total hormone therapy increased. Among the types of prescribed medications, the prescription rate of GnRH analogues decreased, while that of estrogen or progesterone increased sharply, which corresponded to the national health insurance coverage of dienogest from 2013. There was no significant change in the total and medication costs per person, but surgery cost showed a gradual increase.

Laparoscopic treatment of endometriosis is recommended because it leads to improvement in the disease and disease-associated pain^8,26^. According to the ESHRE guidelines for endometriosis, when performing surgery, clinicians may consider excision instead of endometriosis ablation to reduce endometriosis-related pain. When performing surgery in women with ovarian endometrioma, since cystectomy has the advantages of reducing recurrence of endometrioma and endometriosis-associated pain, this procedure should be performed instead of drainage and coagulation. Additionally, clinicians may consider performing surgical removal of deep endometriosis since the technique may reduce endometriosis-associated pain and improve patients’ quality of life^1^. According to the American Society for Reproductive Medicine (ASRM), endometriosis is a chronic condition that requires life-long management. Since surgery has inherent risks and also might result in adhesions that cause pelvic pain and decreased ovarian reserve, a careful strategy that minimizes the use of multiple surgical procedures is needed^12^. In line with the recommendations in the aforementioned guidelines, surgery is considered gradually decreasing and replaced by total hormone therapy for endometriosis treatment.

Dienogest is a fourth-generation selective progestin with the combined pharmacological effects of 19-nortestosterone and progesterone derivatives. This medication shows little androgenic, estrogenic, glucocorticoid or mineralocorticoid activity and minimal adverse effects on metabolic parameters^27^. According to a previous study, dienogest has both anovulatory and antiproliferative effects, while inhibiting the secretion of cytokines in the stroma of endometrial cells^28^. Dienogest was superior to placebo regarding its effect of reducing pelvic pain and showed similar results to those of buserelin, leuprorelin, leuprolide acetate and triptorelin in terms of controlling symptoms associated with endometriosis. Dienogest was effective in reducing endometrial lesions. The extended therapy using dienogest also showed an improvement in pelvic pain after 24–52 weeks with tolerable side effects^27^.

GnRH agonists can induce a reversible pharmacological menopause, reduce production of gonadotrophins, and inhibit ovulation, thereby reducing ovarian steroidogenesis. However, long-term use of GnRH agonist causes side effects, such as the development of a hypoestrogenic state and a decrease in bone mineral density (BMD)^27^. After 24 weeks of treatment with GnRH agonists, BMD decreased by 4–6%, which was much larger as compared to a decrease of 0.5–2.7% in BMD in women treated with dienogest without add-back therapy. Both drugs induce a hypoestrogenic state, which was reportedly moderate in intensity for dienogest as compared to GnRH agonists, or even with other progestins^29^.

The dienogest is recommended as a first-line therapy in endometriosis^30^. Treatment with GnRH analogues or other progestins is only recommended as a second-line therapy. Comparing the 2010 to the 2019 guidelines, GnRH recommendations have changed in favor of other substances, such as dienogest. These changes are due to the adverse events associated with GnRH, including hot flashes or metabolic abnormalities^30,31^. The trend of GnRH analogue replacement with dienogest was also reported in Germany^23^. In Korea, dienogest was covered by the national health insurance from 2013. Since then, the drug has appeared to replace GnRH analogues.

The average number of outpatient visits ranged from two to three; however, approximately 12–18% of the patients underwent surgery and were hospitalized. This discrepancy in the rates is attributed to the fact that surgery is the typical treatment for endometriosis, whereas outpatient visits are generally intermittent and spaced several months apart because of the nature of the disease. Surgical treatment is a major source of high costs for endometriosis patients. According to a study conducted with 10 participating countries in the European Union, 29% of healthcare costs of endometriosis patients were due to surgery^20^. In the U.S., the costs ranged from $4,289 (for diagnostic laparoscopy) to $11,397^32^. The costs related to admission and surgery for endometriosis treatment showed a gradual increase in our study. However, because their rates decreased over the study period, the effect on the total cost was small. Additionally, the medical expenditure on total hormone therapy per person showed a decrease, which might be explained by the continuous decrease in the price of GnRH agonists and its replacement by dienogest, the cheaper option. As a result, despite the increased use of total hormone therapy, its medical cost per person remained stable.

This study had some limitations. The major limitation was that healthcare examination cost was not included in the analysis. Imaging examinations, such as ultrasound, were not covered by national health insurance during the study period, and thus, were not included. We also could not include results from physical examinations and laboratory tests; thus, medical utilization and cost of related complications or adverse events could not be investigated. In addition, only direct medical costs were analyzed, and other indirect costs, such as productivity loss, could not be included in the analysis. Furthermore, we would have liked to examine sequential patterns of healthcare utilization (e.g., medication use after surgery) but were unable to do so because we used cross-sectional data. Such investigations are possible for a long-term cohort. Finally, it may not be straightforward to conclude that a patient received endometriosis treatment only based on the diagnosis code^33^. However, defining endometriosis based on the diagnosis code alone was commonly employed in previous studies^5,22,34^.

Nevertheless, to our best knowledge, the findings from this study were significant because it is the first and the most extensive analysis on the long-term trends of medical procedures for endometriosis treatment in South Korea. Our study shows how trends in endometriosis treatment have changed over a 10-year period. In particular, the surgery and GnRH analog prescription rates decreased and the dienogest prescription rate increased. Thus, the total costs remained stable over the 10-year period. This illustrates how the introduction of new drugs and clinical guidelines affect the clinical field and public health. Since there have only been a few analyses of trends in endometriosis treatment, the results of this study can be used to develop a clinical guideline and build a national health policy in the future.

## Supplementary Information

Supplementary Tables.

## Data Availability

The HIRA-NPS is provided by the Health Insurance Service & Assessment Service in Korea. To protect privacy, access to the data is available only for certified researchers in South Korea. To access the data, application can be made at the following link: https://opendata.hira.or.kr. Detailed information can be found at the same link.
