# Supplementary material for: Current treatments for endometriosis in South Korea: an analysis of nationwide data from 2010 to 2019
Source: Sci Rep. 2023 Jun 13;13:9573. doi: 10.1038/s41598-023-36291-1 (PMC10264383; doi:10.1038/s41598-023-36291-1)
Supplement: Supplementary file 1 — Supplementary Tables. [file 41598_2023_36291_MOESM1_ESM.docx]

**Current treatments for endometriosis in South Korea: an analysis of nationwide data from 2010 to 2019**

Han Kyul Kim^1^, Eun-San Kim^2^, Kyoung Sun Park^1^, Yoon Jae Lee^2^, In-Hyuk Ha^2^*

^1^ Jaseng Hospital of Korean Medicine, Gangnam-daero, Gangnam-gu, Seoul, Republic of Korea

^2^ Jaseng Spine and Joint Research Institute, Jaseng Medical Foundation, Gangnam-daero, Gangnam-gu, Seoul, Republic of Korea

**Supplementary Table S1.** Codes of the treatment definitions.

We analyzed all types of treatment prescribed for endometriosis. Prescribed medications were classified according to the Anatomical Therapeutic Chemical Classification System (ATC code). In this table, only the codes noted in the manuscript were presented. If surgery and prescription rates of total hormone therapy were too low, they were not presented in the manuscript (e.g., aromatase, GnRH antagonists). GnRH, gonadotrophin-releasing hormone; NSAIDs, non-steroidal anti-inflammatory drugs.

| **Drug** | **Code** |
| --- | --- |
| Laparoscopy | Procedure code: N0031001  Diagnosis-related group code: N041, N042, N045, N046, N047, N048 |
| Extirpation of benign adnexal tumor | Procedure code: R4421 |
| Pelviscopic fulguration | Procedure code: R4165 |
| Total hormone therapy | G03, L02, H01CC01, H01CC02 |
| Estrogen or progesterone | G03AA, G03AB, G03AC, G03C, G03D, G03F,  L02AA, L02AB |
| Dienogest | G03DB08 |
| Tibolone | G03CX01 |
| GnRH analogues | G03GA, L02AE, L02BX02, H01CC01, H01CC02 |
| Leuprorelin | L02AE02 |
| Goserelin | L02AE03 |
| NSAIDs | M01A, N02BA |

**Supplementary Table S2.** The distribution of healthcare service utilization by year for patients aged under 40 years.

|  | 2010 | 2011 | 2012 | 2013 | 2014 | 2015 | 2016 | 2017 | 2018 | 2019 | RR (Crude) | RR(Adjusted) |
| --- | --- | --- | --- | --- | --- | --- | --- | --- | --- | --- | --- | --- |
| Outpatient visits | 2.38 (2.14) | 2.62 (2.12) | 2.87 (2.45) | 3.23 (3.78) | 3.05 (2.91) | 3.12 (3.17) | 3.24 (4.30) | 2.87 (2.92) | 3.09 (2.31) | 2.96 (2.51) | 1.02  (1.01 to 1.02)*** | 1.01  (1.01 to 1.02)*** |
| Admission rate | 17.8 | 19.9 | 20.3 | 15.4 | 18.4 | 16.4 | 21.5 | 14.8 | 14.7 | 14.3 | 0.97  (0.95 to 0.99)* | 0.97  (0.95 to 0.99)** |
| Surgery rate | 18.2 | 19.7 | 20.9 | 16.1 | 18.8 | 16.2 | 20.3 | 14.4 | 14.9 | 12.8 | 0.96  (0.94 to 0.98)*** | 0.96  (0.94 to 0.98)*** |
| Laparoscopy | 10.8 | 12.3 | 11.8 | 11.1 | 17.8 | 15.5 | 19.3 | 13.5 | 13.3 | 11.3 |  |  |
| Surgery type |  |  |  |  |  |  |  |  |  |  | 1.01  (0.99 to 1.04) | 1.01  (0.99 to 1.04) |
| Extirpation of  benign adnexal tumor | 11.2 | 10.9 | 11.8 | 12.2 | 13.6 | 11.2 | 14.6 | 10.9 | 10.5 | 9.5 | 0.99  (0.96 to 1.01) | 0.99  (0.96 to 1.01) |
| Pelviscopic  fulguration | 1.5 | 1.9 | 1.1 | 2.3 | 2.7 | 2.2 | 2.0 | 1.4 | 1.4 | 0.6 | 0.95  (0.88 to 1.02) | 0.95  (0.88 to 1.02) |
| Prescription rate |  |  |  |  |  |  |  |  |  |  |  |  |
| Hormone therapy | 42.5 | 38.4 | 37.4 | 39.9 | 48.1 | 46.3 | 50.3 | 47.5 | 54.2 | 52.3 | 1.04  (1.02 to 1.05)*** | 1.04  (1.02 to 1.05)*** |
| Estrogen or  progesterone | 14.0 | 12.0 | 11.8 | 21.3 | 36.4 | 35.8 | 38.7 | 36.9 | 43.4 | 43.8 | 1.14  (1.12 to 1.17)*** | 1.14  (1.12 to 1.17)*** |
| Dienogest | 0.0 | 0.0 | 0.0 | 13.6 | 30.8 | 30.0 | 33.9 | 33.0 | 39.9 | 40.6 | 1.28  (1.25 to 1.31)*** | 1.28  (1.25 to 1.31)*** |
| Tibolone | 4.2 | 3.0 | 3.8 | 3.9 | 3.9 | 2.2 | 2.0 | 2.1 | 2.4 | 2.1 | 0.92  (0.87 to 0.98)** | 0.92  (0.87 to 0.98)** |
| GnRH analogues | 37.0 | 34.0 | 34.5 | 27.9 | 22.1 | 19.0 | 19.1 | 17.2 | 19.8 | 16.9 | 0.91  (0.89 to 0.93)*** | 0.91  (0.89 to 0.93)*** |
| Leuprorelin | 27.3 | 24.5 | 26.5 | 21.1 | 15.3 | 14.9 | 14.4 | 14.6 | 16.3 | 14.4 | 0.92  (0.90 to 0.95)*** | 0.92  (0.90 to 0.95)*** |
| Goserelin | 5.2 | 5.3 | 5.8 | 4.8 | 3.5 | 2.8 | 2.0 | 1.4 | 2.3 | 1.0 | 0.84  (0.80 to 0.89)*** | 0.84  (0.80 to 0.89)*** |
| NSAIDs | 26.5 | 29.4 | 27.4 | 15.2 | 12.0 | 10.3 | 12.2 | 11.6 | 9.6 | 12.8 | 0.88  (0.86 to 0.90)*** | 0.88  (0.86 to 0.91)*** |
| Prescription days |  |  |  |  |  |  |  |  |  |  |  |  |
| Hormone therapy | 18.9 (37.5) | 24.7 (47.8) | 18.1 (34.9) | 42.9 (81.7) | 75.3 (110.7) | 63.4 (95.9) | 72.6 (122.8) | 78.1 (112.6) | 78.8 (121.1) | 71.1 (110.5) | 1.13  (1.13 to 1.13)*** | 1.13  (1.13 to 1.13)*** |
| Estrogen or  progesterone | 46.4 (50.9) | 62 (63.7) | 47.9 (47.3) | 75.7 (99.6) | 97.5 (118.5) | 80.4 (102.6) | 92.3 (133.5) | 98.6 (119.6) | 96.7 (128.9) | 83.5 (116.3) | 1.04  (1.04 to 1.04)*** | 1.04  (1.04 to 1.04)*** |
| Dienogest | — | — | — | 74.3 (108.2) | 102 (124.3) | 84.4 (108.9) | 93.2 (133.9) | 102.7 (124) | 98.5 (128.7) | 85.3 (119.8) | 1.00  (1.00 to 1.00) | 1.00  (1.00 to 1.00) |
| Tibolone | 51.6 (24.8) | 74.5 (47.7) | 68.6 (42.6) | 92.7 (66) | 72.1 (53.2) | 67.9 (46.4) | 70.6 (47.7) | 95 (62.6) | 50.1 (31.4) | 62.9 (41.3) | 1.00  (0.99 to 1.01) | 1.01  (1.00 to 1.02)** |
| GnRH analogues | 3 (1.7) | 3.2 (1.7) | 3.2 (1.8) | 3.4 (1.7) | 3.2 (1.7) | 3.3 (1.8) | 3.4 (1.6) | 3 (1.6) | 3.1 (2) | 3.2 (1.8) | 1.00  (0.99 to 1.01) | 1.00  (0.99 to 1.01) |
| Leuprorelin | 3 (1.7) | 3.2 (1.7) | 3.3 (1.8) | 3.4 (1.7) | 3.1 (1.7) | 3.4 (1.9) | 3.3 (1.6) | 2.9 (1.5) | 3 (1.5) | 3.2 (1.8) | 1.00  (0.99 to 1.01) | 1.00  (0.99 to 1.01) |
| Goserelin | 2.3 (1.5) | 3.2 (1.6) | 2.8 (1.8) | 3.3 (2) | 3.5 (1.4) | 3 (1.5) | 3.7 (1.5) | 3.5 (2.1) | 2.3 (1.7) | 4 (1.3) | 1.02  (0.99 to 1.06) | 1.02  (0.99 to 1.06) |
| NSAIDs | 8.9 (8.1) | 12.6 (20.8) | 10.5 (12) | 14.6 (47.9) | 10.9 (13.7) | 8.5 (7.7) | 12.5 (23.4) | 9.8 (13.8) | 9.8 (11.7) | 11.7 (16) | 1.00  (0.99 to 1.01) | 1.00  (0.99 to 1.01) |
| Outpatient visits and prescription days were provided with the mean (standard deviation) per one patient by year. Prevalence rates were provided with per 100 patients by the year. Prescription days were calculated for patients who were prescribed corresponding medication. Relative ratio (RR) was estimated with Poisson regression. We presented crude and age-adjusted RR per one year. RR of prescription days for dienogest was estimated with data after 2013. *P<0.05; **P<0.01; ***P<0.001. GnRH, gonadotrophin-releasing hormone; NSAIDs, non-steroidal anti-inflammatory drugs. RR, relative ratio. | | | | | | | | | | | | |

**Supplementary Table S3.** The distribution of healthcare service utilization by year for patients aged more than 40 years.

|  | 2010 | 2011 | 2012 | 2013 | 2014 | 2015 | 2016 | 2017 | 2018 | 2019 | RR (Crude) | RR(Adjusted) |
| --- | --- | --- | --- | --- | --- | --- | --- | --- | --- | --- | --- | --- |
| Outpatient visits | 2.40 (2.33) | 2.20 (2.46) | 2.50 (2.14) | 2.79 (3.05) | 2.78 (2.92) | 2.49 (2.34) | 2.45 (2.25) | 2.81 (3.05) | 2.76 (2.50) | 2.58 (1.94) | 1.01  (1.00 to 1.02)** | 1.01  (1.00 to 1.02)** |
| Admission rate | 11.4 | 13.0 | 12.9 | 11.4 | 10.6 | 11.7 | 9.3 | 9.2 | 11.7 | 9.7 | 0.98  (0.94 to 1.02) | 0.98  (0.94 to 1.02) |
| Surgery rate | 12.4 | 15.0 | 15.8 | 16.0 | 15.4 | 16.0 | 12.8 | 12.5 | 12.6 | 12.4 | 0.98  (0.95 to 1.02) | 0.98  (0.95 to 1.02) |
| Laparoscopy | 6.4 | 8.7 | 9.4 | 10.0 | 10.1 | 11.3 | 8.6 | 8.3 | 9.5 | 7.8 | 1.00  (0.96 to 1.05) | 1.00  (0.96 to 1.05) |
| Surgery type |  |  |  |  |  |  |  |  |  |  | 1.03  (0.97 to 1.09) | 1.03  (0.97 to 1.09) |
| Extirpation of  benign adnexal tumor | 5.4 | 4.8 | 5.9 | 5.5 | 6.6 | 6.6 | 5.9 | 5.8 | 7.4 | 6.5 | 1.05  (0.87 to 1.27) | 1.05  (0.87 to 1.28) |
| Pelviscopic  fulguration | 0.5 | 0.5 | 0.0 | 0.0 | 0.4 | 1.2 | 0.7 | 0.8 | 0.6 | 0.3 | 1.01  (1.00 to 1.02)** | 1.01  (1.00 to 1.02)** |
| Prescription rate |  |  |  |  |  |  |  |  |  |  |  |  |
| Hormone therapy | 39.1 | 37.2 | 31.2 | 40.2 | 41.0 | 44.7 | 38.6 | 45.0 | 43.7 | 45.7 | 1.03  (1.00 to 1.05)* | 1.03  (1.00 to 1.05)* |
| Estrogen or  progesterone | 19.8 | 18.8 | 12.4 | 26.9 | 29.5 | 30.7 | 30.0 | 36.2 | 34.8 | 37.0 | 1.09  (1.06 to 1.12)*** | 1.09  (1.06 to 1.12)*** |
| Dienogest | 0.0 | 0.0 | 0.0 | 9.1 | 20.7 | 21.8 | 19.7 | 25.4 | 25.8 | 27.6 | 1.27  (1.22 to 1.32)*** | 1.28  (1.22 to 1.33)*** |
| Tibolone | 4.5 | 7.2 | 4.0 | 5.5 | 2.6 | 3.9 | 2.8 | 3.3 | 3.4 | 3.5 | 0.94  (0.88 to 1.00) | 0.94  (0.88 to 1.00) |
| GnRH analogues | 26.7 | 26.1 | 24.3 | 24.2 | 16.3 | 19.5 | 13.8 | 14.2 | 15.7 | 15.4 | 0.93  (0.90 to 0.96)*** | 0.93  (0.90 to 0.96)*** |
| Leuprorelin | 14.9 | 18.4 | 17.8 | 18.7 | 12.8 | 16.0 | 12.4 | 11.2 | 13.2 | 14.1 | 0.97  (0.93 to 1.00)* | 0.97  (0.93 to 1.00)* |
| Goserelin | 6.9 | 5.8 | 5.4 | 3.7 | 2.2 | 1.2 | 0.7 | 1.7 | 1.5 | 0.5 | 0.77  (0.70 to 0.84)*** | 0.77  (0.70 to 0.84)*** |
| NSAIDs | 26.7 | 26.6 | 25.7 | 21.5 | 16.3 | 11.3 | 13.1 | 10.0 | 12.6 | 13.0 | 0.90  (0.87 to 0.93)*** | 0.90  (0.87 to 0.93)*** |
| Prescription days |  |  |  |  |  |  |  |  |  |  |  |  |
| Hormone therapy | 35.2 (73.5) | 38.9 (77.8) | 39.3 (76.8) | 56.8 (89.3) | 81 (113.5) | 62.4 (100.6) | 68.6 (103.5) | 69.6 (114.2) | 77.5 (114.5) | 79.5 (115.7) | 1.08  (1.08 to 1.08)*** | 1.08  (1.08 to 1.09)*** |
| Estrogen or  progesterone | 59.8 (94.4) | 72.8 (97.2) | 93.6 (99.6) | 80.8 (99.7) | 109 (121.9) | 88.5 (111.6) | 86.8 (111) | 74.6 (115.5) | 93.1 (121.8) | 96.6 (122) | 1.02  (1.02 to 1.03)*** | 1.03  (1.03 to 1.03)*** |
| Dienogest | — | — | — | 85.6 (103.9) | 119.1 (124.6) | 74.8 (104.5) | 92.3 (114.5) | 64.6 (93.8) | 76.3 (107.3) | 87.9 (113.2) | 0.97  (0.96 to 0.97)*** | 0.97  (0.96 to 0.97)*** |
| Tibolone | 56.6 (63.7) | 101.3 (94.3) | 126.2 (100.4) | 149.3 (125.3) | 127.8 (131.6) | 128.8 (129.7) | 118.8 (115.2) | 113.4 (149.1) | 182.6 (114.9) | 141.1 (131.4) | 1.06  (1.05 to 1.06)*** | 1.06  (1.06 to 1.07)*** |
| GnRH analogues | 2.8 (1.8) | 2.6 (1.8) | 2.8 (1.8) | 3.3 (1.7) | 2.9 (1.7) | 2.6 (1.8) | 2.5 (1.6) | 2.6 (1.4) | 3.2 (1.7) | 2.6 (1.5) | 1.00  (0.98 to 1.02) | 1.00  (0.98 to 1.02) |
| Leuprorelin | 3.1 (1.8) | 2.1 (1.5) | 2.6 (1.8) | 3.2 (1.6) | 3.1 (1.8) | 2.6 (1.8) | 2.6 (1.6) | 2.4 (1.2) | 3 (1.6) | 2.7 (1.5) | 1.00  (0.98 to 1.02) | 1.00  (0.98 to 1.02) |
| Goserelin | 2.6 (1.9) | 3.5 (2.1) | 2.6 (1.5) | 2.8 (1.9) | 2 (1.2) | 1.7 (1.2) | 2 (1.4) | 3.2 (2.1) | 4.4 (1.8) | 1.5 (0.7) | 1.01  (0.95 to 1.06) | 1.01  (0.95 to 1.06) |
| NSAIDs | 15.9 (27.7) | 20.4 (46.4) | 7.4 (5.4) | 12.8 (16.2) | 6.9 (7.3) | 14.2 (22.1) | 8.7 (6.2) | 6.2 (6.3) | 10.1 (15.3) | 14.1 (32.9) | 0.95  (0.94 to 0.96)*** | 0.95  (0.94 to 0.96)*** |
| Outpatient visits and prescription days were provided with the mean (standard deviation) per one patient by year. Prevalence rates were provided with per 100 patients by the year. Prescription days were calculated for patients who were prescribed corresponding medication. Relative ratio (RR) was estimated with Poisson regression. We presented crude and age-adjusted RR per one year. RR of prescription days for dienogest was estimated with data after 2013. *P<0.05; **P<0.01; ***P<0.001. GnRH, gonadotrophin-releasing hormone; NSAIDs, non-steroidal anti-inflammatory drugs. RR, relative ratio. | | | | | | | | | | | | |

**Supplementary Table S4.** The distribution of medical costs by year for patients aged under 40 years.

|  | 2010 | 2011 | 2012 | 2013 | 2014 | 2015 | 2016 | 2017 | 2018 | 2019 | Cost ratio  (Crude) | Cost ratio (Adjusted) |
| --- | --- | --- | --- | --- | --- | --- | --- | --- | --- | --- | --- | --- |
| Total cost | 546.5 (817.9) | 563.3 (801.2) | 552.8 (818.1) | 535.4 (879.5) | 683 (1040.2) | 613.3 (949.3) | 733.9 (1091.1) | 581.6 (973.4) | 654 (1032.9) | 580.2 (1039.4) | 1.01  (1.00 to 1.03) | 1.01  (1.00 to 1.03) |
| Outpatient visits | 281.7 (378.5) | 283.2 (359.4) | 226.4 (299.3) | 228.4 (282.5) | 266.9 (330.4) | 250.1 (275.4) | 266.9 (286.8) | 238.9 (261.2) | 290.4 (319.3) | 239 (277.9) | 1.00  (0.98 to 1.01) | 1.00  (0.99 to 1.01) |
| Admission | 1547.5 (355.1) | 1456.4 (417.2) | 1645 (354.8) | 2021 (567.1) | 2292.8 (470.7) | 2263.7 (615.5) | 2213.7 (786.8) | 2350.2 (817) | 2510.6 (786.4) | 2424.6 (1041.2) | 1.06  (1.05 to 1.07)*** | 1.06  (1.05 to 1.07)*** |
| Surgery | 1501.7 (437.1) | 1452.9 (449.3) | 1582.3 (465.8) | 1926 (670.2) | 2192.8 (578.1) | 2253.2 (617.7) | 2271.1 (669.7) | 2393.8 (776.3) | 2477.4 (854.3) | 2596.8 (947.2) | 1.07  (1.06 to 1.08)*** | 1.07  (1.06 to 1.08)*** |
| Hormone therapy | 486.2 (331) | 507.3 (335) | 419 (270.7) | 384.5 (267.8) | 395.9 (330.9) | 358.2 (233.9) | 346.6 (240.5) | 337.2 (216.3) | 362.9 (224.1) | 267.6 (183.4) | 0.95  (0.94 to 0.96)*** | 0.95  (0.94 to 0.96)*** |
| Estrogen or  progesterone | 18 (18.4) | 26.9 (23.1) | 20.7 (24.3) | 172.3 (188.2) | 293.2 (317.3) | 268.3 (211.9) | 280 (223.7) | 288.4 (195.3) | 312.1 (208.4) | 197.8 (138.8) | 1.14  (1.12 to 1.17)*** | 1.14  (1.12 to 1.17)*** |
| Dienogest | — | — | — | 257.2 (183.9) | 342.5 (320.4) | 317.2 (196.5) | 316.1 (213.5) | 319.6 (181.6) | 337.6 (196.2) | 212.6 (132.8) | 0.95  (0.93 to 0.97)*** | 0.95  (0.93 to 0.97)*** |
| Tibolone | 19.2 (9.3) | 25.9 (16.3) | 19.8 (11.5) | 24.9 (18) | 18.9 (13.9) | 17.5 (12) | 17.3 (12.6) | 22.3 (14.9) | 10.8 (6.4) | 11.6 (8.1) | 0.94  (0.90 to 0.97)** | 0.94  (0.91 to 0.98)** |
| GnRH analogues | 550.2 (294.2) | 558.9 (302.7) | 446.8 (250.5) | 418.5 (243.7) | 379.9 (228.4) | 369.1 (211.3) | 345 (200.3) | 309.6 (197.4) | 307.4 (195.9) | 314.3 (185.6) | 0.93  (0.92 to 0.94)*** | 0.93  (0.92 to 0.94)*** |
| Leuprorelin | 559.3 (299.1) | 566.7 (307.2) | 426.7 (213) | 369.6 (186.4) | 315.2 (166.1) | 341.8 (184.8) | 294.8 (143.1) | 273.5 (142.1) | 281 (141.8) | 295.8 (163) | 0.92  (0.91 to 0.93)*** | 0.92  (0.91 to 0.93)*** |
| The costs were provided with the mean (standard deviation) per one patient by year. Costs were calculated for patients who used corresponding medical services. Cost ratio was estimated with generalized linear regression with log-link gamma distribution. We presented crude and age-adjusted cost ratio per one year. Cost ratio for dienogest change was estimated with data after 2013. *P<0.05; **P<0.01; ***P<0.001. GnRH, gonadotrophin-releasing hormone; NSAIDs, non-steroidal anti-inflammatory drugs. | | | | | | | | | | | | |

**Supplementary Table S5.** The distribution of medical costs by year for patients aged more than 40 years.

|  | 2010 | 2011 | 2012 | 2013 | 2014 | 2015 | 2016 | 2017 | 2018 | 2019 | Cost ratio  (Crude) | Cost ratio (Adjusted) |
| --- | --- | --- | --- | --- | --- | --- | --- | --- | --- | --- | --- | --- |
| Total cost | 438.9 (763.7) | 404.3 (644.6) | 419.2 (730.6) | 439.2 (827.3) | 436.5 (732.9) | 458.5 (827.3) | 372.5 (706) | 417.3 (784.4) | 517.2 (1114.6) | 438.4 (886.3) | 1.01  (0.98 to 1.04) | 1.01  (0.98 to 1.04) |
| Outpatient visits | 248.4 (379) | 212.4 (312.3) | 180.2 (246.4) | 208.1 (259.3) | 213.6 (255.4) | 199.1 (235.9) | 171.4 (213.6) | 210.3 (251.7) | 215.2 (250.8) | 188.6 (206.1) | 0.99  (0.97 to 1.00) | 0.98  (0.97 to 1.00) |
| Admission | 1694.4 (762.1) | 1549.7 (627.2) | 1899 (685.3) | 2058.3 (810.5) | 2161.5 (428.9) | 2281.7 (547.2) | 2197.8 (622.2) | 2306.3 (779.3) | 2627.6 (1815.4) | 2635.7 (1006.8) | 1.06  (1.04 to 1.07)*** | 1.06  (1.04 to 1.07)*** |
| Surgery | 1521.6 (905.3) | 1355.4 (772) | 1474.1 (984.9) | 1418.7 (1004) | 1498.2 (1021.2) | 1697.5 (1078.5) | 1584.4 (1035.9) | 1730.4 (1177.2) | 2362 (1655.2) | 1912 (1435.9) | 1.05  (1.02 to 1.07)*** | 1.05  (1.02 to 1.08)*** |
| Hormone therapy | 392.2 (384.9) | 324.4 (326.4) | 316.2 (278.6) | 307.9 (249.7) | 320.6 (253.8) | 274.8 (213.6) | 248.7 (215.5) | 267.9 (230.8) | 288.7 (230.2) | 211.9 (170.4) | 0.95  (0.94 to 0.97)*** | 0.95  (0.94 to 0.97)*** |
| Estrogen or  progesterone | 27.2 (32.9) | 36.2 (35.1) | 28.9 (27.2) | 100.4 (142.5) | 252.1 (227.6) | 223.4 (199.7) | 195.2 (194) | 219.8 (200.8) | 203.5 (160.9) | 152.5 (136.9) | 1.15  (1.12 to 1.19)*** | 1.14  (1.10 to 1.17)*** |
| Dienogest | — | — | — | 241.3 (169) | 347.4 (205.9) | 298.3 (189.3) | 285.4 (180.9) | 298.1 (186.3) | 260.9 (145.4) | 195.2 (131.8) | 0.93  (0.90 to 0.96)*** | 0.93  (0.90 to 0.96)*** |
| Tibolone | 28.1 (26.5) | 37.4 (34.3) | 37.5 (28.6) | 41.1 (34.5) | 33.4 (34.4) | 33.3 (33.5) | 30.4 (29.5) | 26.6 (30) | 40.2 (25.9) | 26.1 (23.6) | 0.98  (0.93 to 1.04) | 0.99  (0.93 to 1.05) |
| GnRH analogues | 545.1 (359.9) | 435.5 (309.3) | 391.8 (261.6) | 399 (229) | 329.8 (224.6) | 275.3 (176.4) | 261.8 (165.2) | 270.3 (199.4) | 350.4 (213.6) | 258.5 (144.1) | 0.93  (0.92 to 0.95)*** | 0.93  (0.92 to 0.95)*** |
| Leuprorelin | 622.8 (345.2) | 348.3 (233.6) | 337.9 (232) | 356.3 (171.8) | 333.4 (246.7) | 263 (167.8) | 259.4 (163.6) | 227.5 (116.9) | 300.7 (153.1) | 254.2 (142) | 0.93  (0.91 to 0.95)*** | 0.93  (0.91 to 0.95)*** |
| The costs were provided with the mean (standard deviation) per one patient by year. Costs were calculated for patients who used corresponding medical services. Cost ratio was estimated with generalized linear regression with log-link gamma distribution. We presented crude and age-adjusted cost ratio per one year. Cost ratio for dienogest change was estimated with data after 2013. *P<0.05; **P<0.01; ***P<0.001. GnRH, gonadotrophin-releasing hormone; NSAIDs, non-steroidal anti-inflammatory drugs. | | | | | | | | | | | | |
